# Author Correction: Decreased integrity of exercise-induced plasma cell free nuclear DNA – negative association with the increased oxidants production by circulating phagocytes

**DOI:** 10.1038/s41598-020-68815-4

**Published:** 2020-07-13

**Authors:** Robert Stawski, Konrad Walczak, Ewelina Perdas, Anna Wlodarczyk, Agata Sarniak, Piotr Kosielski, Pawel Meissner, Tomasz Budlewski, Gianluca Padula, Dariusz Nowak

**Affiliations:** 1 Department of Clinical Physiology, Medical University of Lodz, Lodz, Poland; 2 Department of Internal Medicine and Nephrodiabetology, Medical University of Lodz, Lodz, Poland; 3 Department of Cardiovascular Physiology, Faculty of Medicine, Medical University of Lodz, Lodz, Poland; 4 Department of Sleep Medicine and Metabolic Disorders, Medical University of Lodz, Lodz, Poland; 5 Department of General Physiology, Medical University of Lodz, Lodz, Poland; 6 Academic Laboratory of Movement and Human Physical Performance, Medical University of Lodz, Lodz, Poland; 7 Department of Rheumatology, Medical University of Lodz, University Hospital Name of the Military Medical Academy-Central Hospital Veterans of Lodz, Ul. Pieniny 30, 92-115, Łódź , Poland

Correction to: *Scientific Reports* https://doi.org/10.1038/s41598-019-52409-w, published online 04 November 2019

In the original version of this Article, Tomasz Budlewski was incorrectly affiliated with ‘Department of Internal Medicine, University Hospital name of the Military Medical Academy Central Hospital Veterans of Lodz, Lodz, Poland’.

The correct affiliation is listed below.

Department of Rheumatology, Medical University of Lodz, University Hospital name of the Military Medical Academy-Central Hospital Veterans of Lodz Ul. Pieniny 30, 92-115 Łódź, Poland.

This error has now been corrected in the PDF and HTML versions of the Article.

