# Supplementary material for: Decreased integrity of exercise-induced plasma cell free nuclear DNA – negative association with the increased oxidants production by circulating phagocytes
Source: Sci Rep. 2019 Nov 4;9:15970. doi: 10.1038/s41598-019-52409-w (PMC6828751; doi:10.1038/s41598-019-52409-w)
Supplement: Supplementary file 1 — Supplementary information [file 41598_2019_52409_MOESM1_ESM.docx]

**Supplementary material for**

**Decreased integrity of exercise-induced plasma cell free nuclear DNA – negative**

**association with the increased oxidants production by circulating phagocytes**

Robert Stawski, Konrad Walczak, Ewelina Perdas, Anna Wlodarczyk, Agata Sarniak, Piotr Kosielski,

Pawel Meissner, Tomasz Budlewski, Gianluca Padula, Dariusz Nowak

| **Table 5. Cell free nuclear DNA (cf n-DNA) integrity in eleven average-trained men before and after each of three bouts of exhaustive treadmill exercise.** | | | | | | | | | | | |  |
| --- | --- | --- | --- | --- | --- | --- | --- | --- | --- | --- | --- | --- |
|  |  |  |  |  | |  |  |  | |  | |  |
| **cf n-DNA integrity** | 1^st^ bout |  |  | 2^nd^ bout | |  |  | 3^rd^ bout | |  | |  |
|  | before | after |  | before | | after |  | before | | after | |  |
|  | 0.571 | 0.523 |  | 0.863 | | 0.745 |  | 0.336 | | 0.316 | |  |
|  | 0.336 | 0.243 |  | 0.571 | | 0.464 |  | 0.378 | | 0.378 | |  |
|  | 0.744 | 0.209 |  | 0.554 | | 0.438 |  | 0.316 | | 0.307 | |  |
|  | 0.507 | 0.413 |  | 0.367 | | 0.236 |  | 0.367 | | 0.236 | |  |
|  | 0.790 | 0.464 |  | 0.571 | | 0.571 |  | 0.316 | | 0.316 | |  |
|  | 0.745 | 0.662 |  | 0.571 | | 0.554 |  | 0.943 | | 0.507 | |  |
|  | 0.943 | 0.767 |  | 0.538 | | 0.464 |  | 0.681 | | 0.571 | |  |
|  | 0.662 | 0.588 |  | 0.554 | | 0.378 |  | 0.186 | | 0.538 | |  |
|  | 0.451 | 0.745 |  | 0.523 | | 0.642 |  | 0.538 | | 0.889 | |  |
|  | 0.367 | 0.367 |  | 0.281 | | 0.203 |  | 0.642 | | 0.493 | |  |
|  | 0.326 | 0.298 |  | 0.438 | | 0.175 |  | 0.789 | | 0.413 | |  |
|  |  |  |  |  | |  |  |  | |  | |  |
| **Table 6. Cell free mitochondrial DNA (cf mt-DNA) integrity in eleven average-trained men before and after each of three bouts of exhaustive treadmill exercise.** | | | | | | | | | | | |  |
|  |  |  |  |  | |  |  | |  | |  | |
| **cf mt-DNA integrity** | 1^st^ bout |  |  | 2^nd^ bout | |  |  | | 3^rd^ bout | |  | |
|  | before | after |  | before | | after |  | | before | | after | |
|  | 0.594 | 0.432 |  | 0.419 | | 0.544 |  | | 0.943 | | 0.971 | |
|  | 0.749 | 0.771 |  | 0.816564 | | 0.817 |  | | 0.667 | | 0.944 | |
|  | 0.209 | 0.181 |  | 0.666777 | | 0.971 |  | | 0.630 | | 0.514 | |
|  | 0.560 | 0.707 |  | 0.943744 | | 0.499 |  | | 0.793 | | 0.667 | |
|  | 0.648 | 0.686 |  | 0.52893 | | 0.629 |  | | 0.817 | | 0.458 | |
|  | 0.323 | 0.594 |  | 0.48493 | | 0.611 |  | | 0.667 | | 0.296 | |
|  | 0.419 | 0.817 |  | 0.45765 | | 0.514 |  | | 0.629 | | 0.793 | |
|  | 0.445 | 0.891 |  | 0.499174 | | 0.629 |  | | 0.471 | | 0.793 | |
|  | 0.707 | 0.971 |  | 0.64775 | | 0.817 |  | | 0.648 | | 0.865 | |
|  | 0.594 | 0.419 |  | 0.943744 | | 0.891 |  | | 0.707 | | 0.105 | |
|  | 0.727 | 0.111 |  | 0.52893 | | 0.471 |  | | 0.181 | | 0.817 | |
|  |  |  |  |  | |  |  | |  | |  | |
|  |  |  |  |  | |  |  | |  | |  | |
|  |  |  |  |  | |  |  | |  | |  | |
| **Tabel 7. Resting (rLBCL) whole blood chemiluminescence in eleven average-trained men before and after each of three bouts of exhaustive treadmill exercise.** | | | | | | | | | | | |  |
|  |  |  |  |  | |  |  |  | |  | |  |
| **rLBCL** | 1^st^ bout |  |  | 2^nd^ bout | |  |  | 3^rd^ bout | |  | |  |
|  | before | after |  | before | | after |  | before | | after | |  |
|  | 83.60 | 127.31 |  | 41.71 | | 114.40 |  | 31.21 | | 16.60 | |  |
|  | 196.78 | 793.33 |  | 194.89 | | 728.25 |  | 137.52 | | 429.06 | |  |
|  | 61.52 | 578.04 |  | 280.36 | | 305.51 |  | 104.42 | | 184.07 | |  |
|  | 895.93 | 1116.67 |  | 750.36 | | 2153.24 |  | 678.94 | | 1779.17 | |  |
|  | 563.41 | 951.19 |  | 421.19 | | 1171.48 |  | 759.65 | | 1309.47 | |  |
|  | 154.18 | 214.56 |  | 58.05 | | 120.38 |  | 47.96 | | 112.20 | |  |
|  | 32.68 | 653.74 |  | 46.85 | | 291.56 |  | 42.72 | | 362.88 | |  |
|  | 326.00 | 988.46 |  | 175.75 | | 367.31 |  | 534.37 | | 944.09 | |  |
|  | 688.03 | 935.60 |  | 609.15 | | 1308.47 |  | 326.44 | | 888.49 | |  |
|  | 185.58 | 519.82 |  | 638.30 | | 1661.43 |  | 576.42 | | 577.02 | |  |
|  | 396.85 | 786.11 |  | 473.74 | | 1348.26 |  | 274.55 | | 1812.95 | |  |
|  |  |  |  |  | |  |  |  | |  | |  |
|  |  |  |  |  | |  |  |  | |  | |  |
|  |  |  |  |  | |  |  |  | |  | |  |
| **Table 8. fMLP-stimulated luminol enhanced whole blood chemiluminescence (fMLP-LBCL) in eleven average-trained men before and after each of three bouts of exhaustive treadmill exercise.** | | | | | | | | | | | |  |
|  |  |  |  | |  |  |  |  | |  | |  |
| **fMLP-LBCL** | 1^st^ bout |  |  | | 2^nd^ bout |  |  | 3^rd^ bout | |  | |  |
|  | before | after |  | | before | after |  | before | | after | |  |
|  | 447.02 | 737.44 |  | | 363.24 | 497.067 |  | 302.93 | | 353.77 | |  |
|  | 849.66 | 2011.14 |  | | 734.56 | 1613.33 |  | 802.48 | | 921.03 | |  |
|  | 1008.56 | 1745.33 |  | | 1361.32 | 1120.95 |  | 1314.24 | | 951.16 | |  |
|  | 1430.57 | 1401.73 |  | | 1413.33 | 2810.00 |  | 1446.97 | | 2326.15 | |  |
|  | 955.83 | 1444.88 |  | | 642.94 | 1649.73 |  | 1168.07 | | 1764.73 | |  |
|  | 942.49 | 833.68 |  | | 885.53 | 1030.98 |  | 567.87 | | 883.90 | |  |
|  | 169.59 | 1575.38 |  | | 334.72 | 505.46 |  | 318.16 | | 910.07 | |  |
|  | 826.00 | 2846.97 |  | | 1335.33 | 1483.68 |  | 2149.11 | | 3095.91 | |  |
|  | 1806.41 | 1744.26 |  | | 1295.81 | 2087.08 |  | 1140.76 | | 1655.80 | |  |
|  | 430.47 | 656.07 |  | | 725.44 | 2553.20 |  | 663.41 | | 427.93 | |  |
|  | 941.80 | 1848.52 |  | | 903.94 | 2223.41 |  | 682.63 | | 2883.85 | |  |

**Table 9. Exact p-values for statistical comparison of integrity index of cf n-DNA (I_229/97_) and cf mt-DNA (I_218/78_) before and after each of three bouts of exhaustive treadmill exercise in eleven average trained men .**

| Parameter | 1^st^ bout | N=11 |  | 2^nd^ bout | N=11 |  | 3^rd^ bout | N=11 |  | Total | N=33 |
| --- | --- | --- | --- | --- | --- | --- | --- | --- | --- | --- | --- |
|  | before | after |  | before | after |  | before | after |  | before | after |
| **cf n-DNA (I_229/97_)** | **0.046** | |  | **0.036** | |  | 0.26 | |  | **0.0058** | |
| **cf mt-DNA (I_218/78_)** | 0.8 | |  | 0.16 | |  | 0.92 | |  | 0.3 | |

**Table 10. Exact p-values for statistical comparison of resting luminol enhanced whole blood chemiluminescence (rLBCL) and fMLP-stimulated luminol enhanced whole blood chemiluminescence (fMLP-LBCL) before and after each of three bouts of exhaustive treadmill exercise in eleven average trained men .**

| Parameter | 1^st^ bout | N=11 |  | 2^nd^ bout | N=11 |  | 3^rd^ bout | N=11 |  | Total | N=33 |
| --- | --- | --- | --- | --- | --- | --- | --- | --- | --- | --- | --- |
|  | before | after |  | before | after |  | before | after |  | before | after |
| **rLBCL** | **0.0033** | |  | **0.0032** | |  | **0.0058** | |  | **0.000001** | |
| **fMLP-LBCL** | **0,0163** | |  | **0.0127** | |  | **0.026** | |  | **0.000026** | |
